# Elevated Serum IL-17 Expression at Cessation Associated with Graves' Disease Relapse

**DOI:** 10.1155/2018/5689030

**Published:** 2018-03-11

**Authors:** Jianhui Li, Xiaohua Sun, Danzhen Yao, Jinying Xia

**Affiliations:** Department of Endocrine, Ningbo No. 2 Hospital, No. 41, Xibei Street, Ningbo, 315000 Zhejiang, China

## Abstract

**Background:**

Antithyroid drug (ATD) treatment occupies the cornerstone therapeutic modality of Graves' disease (GD) with a high relapse rate after discontinuation. This study aimed to assess potential risk factors for GD relapse especially serum interleukin-17 (IL-17) expression.

**Methods:**

Consecutive newly diagnosed GD patients who were scheduled to undergo ATD therapy from May 2011 to May 2014 were prospectively enrolled. Risk factors for GD relapse were analyzed by univariate and multivariate Cox proportional hazard analyses. The association between serum IL-17 expression at cessation and GD relapse was analyzed with relapse-free survival (RFS) by the Kaplan–Meier survival analysis and log-rank test.

**Results:**

Of the 117 patients, 72 (61.5%) maintained a remission for 12 months after ATD withdrawal and 45 (38.5%) demonstrated GD relapse. The final multivariate Cox analysis indicated elevated IL-17 expression at cessation to be an independent risk factor for GD relapse within 12 months after ATD withdrawal (HR: 3.04, 95% CI: 1.14–7.67, *p* = 0.021). Patients with higher expressions of IL-17 (≥median value) at cessation demonstrated a significantly higher RFS than those with lower levels by the Kaplan–Meier analysis and log-rank test (*p* = 0.028).

**Conclusions:**

This present study indicated elevated serum IL-17 expression at cessation to be a predictor for GD relapse within 12 months.

## 1. Introduction

Graves' disease (GD), the most common etiology of spontaneous hyperthyroidism worldwide [1], is recognized as an autoimmune thyroid disease and is characterized by overexpressed thyroid hormones stimulated by thyroid-stimulating hormone (TSH) receptor antibodies (TRAb) [2]. Currently, antithyroid drugs (ATDs), thyroidectomy, and radioactive iodine are the primary therapeutic strategies for GD, while ATD treatment occupies the cornerstone therapeutic modality of GD [3]. ATD therapy is well accepted by both patients and clinicians due to its rapid effect, beneficial immunosuppressive effects, and avoidance of invasive procedures [4]. However, the high relapse rate after discontinuation is the main drawback of ATD therapy, which significantly limits its efficacy [5]. The relapse rate after ATD withdrawal varies greatly among patients with different races, characteristics, genetic factors, and therapy strategies and is reportedly as high as 50%–60% within two years especially in the first year [6]. Relapsing GD is usually more severe and accompanied by chronic thyroid dysfunction. Furthermore, relapsing GD always requires more aggressive therapies and finally results in hypothyroidism [7]. Therefore, relapse prediction after ATD withdrawal is critically important for minimizing the relapse rate and relapse-associated harms and supporting therapy strategy choices.

Th17 cells, as a newly discovered CD4^+^ T cell subset, play an important role in inflammatory and immune reactions, mainly by producing IL-17 [8]. Previous studies have revealed the critical role of Th17 cells and IL-17 in various autoimmune diseases, including the pathogenesis of Hashimoto's thyroiditis, GD, and Graves' ophthalmopathy (GO) [9]. However, whether IL-17 expression can predict the relapse of GD remains unclear. In this regard, our current study aimed to assess the potential relationship between IL-17 expression and GD relapse.

## 2. Material and Methods

### 2.1. Patients

This study protocol was approved by the Medical Institutional Ethics Committee of Zhejiang Province and Ningbo No. 2 Hospital. Consecutive newly diagnosed GD patients who were scheduled to undergo ATD therapy from May 2011 to May 2014 were prospectively enrolled in the Department of Endocrinology at Ningbo No. 2 Hospital. Inclusion criteria were described as follows: (1) newly diagnosed with GD according to the results of physical examinations, radionuclide imaging, and laboratory tests; (2) having a duration of follow-up after ATD discontinuation of at least 1 year; (3) with written informed consent; and (4) without medications that might affect thyroid function (e.g., corticosteroids and amiodarone). Those patients who were pregnant or became pregnant during the therapy or follow-up period were excluded. Those who underwent RAI or surgery due to inefficacy or major side effects of ATD therapy were also excluded. Of the 117 enrolled patients, 96 underwent methimazole (MMI) treatment and 21 underwent propylthiouracil (PTU) treatment. The initial dosage of MMI (or PTU) in our study was 30 mg/d (or 300 mg/d); the subsequent dosage was adjusted appropriately to maintain euthyroidism and tapered gradually due to the thyroid function tests. ATD therapy was discontinued if free T4 (fT4) and thyroid-stimulating hormone (TSH) levels remained normal for ≥6 months with the minimum maintenance dose of ATD (MMI 5 mg/d or PTU 50 mg/d), regardless of the status of TRAb. Participants were followed-up at months 3, 6, 9, and 12 after ATD withdrawal.

The following parameters were retrieved from each enrolled participant: age at diagnosis, gender, smoking habits, presence of Graves' ophthalmopathy, thyroglobulin (Tg), antithyroid peroxidase antibody (anti-TPO), thyroglobulin antibody positivity, ATD drugs, duration of ATD therapy, and time to normalization of TSH and fT4.

### 2.2. Laboratory Tests

The thyroid function tests including fT4, total triiodothyronine (TT3), TSH, TRAb, and thyroid stimulatory antibody (TSAb) were performed at diagnosis and at cessation. The serum expressions of T helper cell cytokines including IFN-*γ* (by Th1 cells), IL-4 (by Th2 cells), and IL-17 (by Th17 cells) were also measured both at diagnosis and at cessation. Serum expressions of fT4, TT3, and Tg antibody titers were measured by the method of radioimmunoassay (Beijing Atom High-Tech, Beijing, China). TSH concentration measurement was conducted with the method of immunoradiometric assay (Beijing Atom High-Tech, Beijing, China). TRAb concentrations were detected with a Roche assay and the Cobas E411 analytical platform (Roche Diagnostics, West Sussex, UK). IFN-*γ*, IL-4, and IL-17 expressions were measured by enzyme-linked immunosorbent assay (ELISA) kits (R&D systems, Minneapolis, MN, USA).

### 2.3. Relapse and Remission Definition

GD relapse and remission in this current study were defined according to our previous reports [10]. In brief, relapse was defined as clinical symptoms of hyperthyroidism with decreased TSH and elevated fT4 (and/or TT3), while remission was defined as clinical and biochemical euthyroidism after drug withdrawal until the end of follow-up.

### 2.4. Statistical Analysis

The statistical analysis was conducted with SPSS 19.0 (SPSS Inc., Chicago, IL, USA) and GraphPad Prism 5.0 (GraphPad Inc., San Diego, CA, USA). The continuous variables were presented as the mean ± SD and compared by Student's *t*-test or Mann–Whitney *U* test. The categorical variables were presented as the numbers with percentages (*n*, %) and compared by the chi-squared test or Fisher's exact test as appropriate. Risk factors for GD relapse within 1 year after ATD discontinuation were analyzed by univariate and multivariate Cox proportional hazard analysis. The relapse-free survival (RFS) curve analysis according to serum IL-17 expression was performed with the Kaplan–Meier method and the log-rank test. A two-sided *p* value < 0.05 was considered statistically different.

## 3. Results

The patient characteristics are illustrated in Table 1. The mean age of all the enrolled 117 patients at diagnosis was 42.0 years, and 91 (77.8%) were females. Of the 117 patients, 72 (61.5%) maintained a remission for 12 months after ATD withdrawal and 45 (38.5%) demonstrated GD relapse. As presented in Table 1, no significant differences in gender (*p* = 0.65), smoking habits (*p* = 0.80), Tg (*p* = 0.45), Tg antibody positivity (*p* = 0.29), duration of ATD therapy (*p* = 0.56), anti-TPO (*p* = 0.085), and ATD drugs (*p* = 0.65) were observed in patients with or without relapse. A younger age at diagnosis (*p* = 0.029), a high presence of Graves' ophthalmopathy (*p* = 0.029), and longer time to normalization of TSH (*p* = 0.007) or fT4 (*p* = 0.003) was significantly associated with an elevated risk of relapse. The serum expressions of thyroid function parameters at diagnosis did not differ between patients with relapse and in remission (*p* = 0.72, 0.16, 1.00, and 0.31, resp.). However, lower duration of remission was observed in patients with higher TRAb (*p* = 0.001) and lower TSH (*p* = 0.013) expressions at cessation.

Serum expression levels of T helper cytokines including IFN-*γ*, IL-4, and IL-17 are presented in Table 2. The serum IL-17 and IL-4 concentrations were significantly higher in GD patients with relapse than they were in remission (*p* = 0.022 and 0.001, resp.).

We constructed Cox proportional hazard regression models including the potential risk factors mentioned above, such as age at diagnosis, presence of Graves' ophthalmopathy, time to normalization of TSH and fT4, and serum expression of TSH, TRAb, IL-4, and IL-17 at cessation. In this model, the final multivariate Cox analysis indicated IL-17 expression at cessation to be an independent risk factor for GD relapse within 12 months after ATD withdrawal (HR: 3.04, 95% CI: 1.14–7.67, *p* = 0.021), which is shown in Table 3.

**Figure 1:**
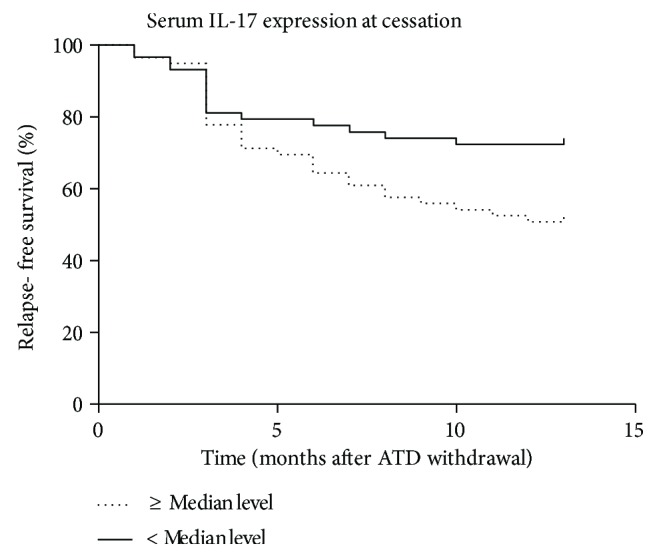
The relapse-free survival (RFS) and serum IL-17 expression at cessation by the Kaplan–Meier survival analysis and log-rank test. Patients with higher expressions of IL-17 (≥median value) at cessation demonstrated a significantly higher RFS than those with lower levels (*p* = 0.028).

The RFS curve analysis according to serum IL-17 expression is illustrated in Figure 1. Patients with higher expression levels of IL-17 (≥median value) at cessation demonstrated a significantly higher RFS than those with lower levels by the Kaplan–Meier analysis and the log-rank test (*p* = 0.028).

## 4. Discussion

We observed an overall relapse rate of 38.5%, which was quite in line with the 37% observed in the study of Vos et al. [5]. To our knowledge, this present study is the first to indicate elevated serum IL-17 expression at cessation as a predictive factor for GD relapse within 12 months after ATD withdrawal. The data in this current study demonstrated elevated IL- 17 levels associated with GD relapse and provided a new insight into the etiology and relapse of GD. Previous studies have revealed some risk factors for GD relapse, such as age, gender, GD family history, and thyroid hormone levels [11, 12]. TRAb positivity has also been reported to be a significant factor associated with GD relapse [13]. However, according to our results by multivariate COX proportional hazard and RFS analysis, IL-17 expression at cessation was the only significant predictor of GD relapse. The findings in the present study did not reconcile with the reports mentioned above but are in agreement with other investigations suggesting no predictive value of TRAbs [14]. The difference in cohort sample size, detection or statistical methods, race, and demographic data might be possible explanations for the discrepancy of conclusions, but further large-scaled investigations are required to clarify the discrepancy. The expression levels of IFN-*γ* at diagnosis or at cessation did not differ between GD patients with and without relapse. Patients with GD relapse had higher IL-4 concentrations at cessation; however, our final results did not support a close association between IL-4 levels and GD relapse. Serum IL-4 expression has been observed to be closely associated with the disease activity of thyroid-associated ophthalmopathy and negatively correlated with TRAb levels [15]. As reported in previous studies, elevated IFN-*γ* and decreased IL-4 levels are observed in GD patients in comparison with healthy controls, which indicates that the Th1/Th2 imbalance may be implicated in the pathogenesis of GD [16]. To our knowledge, whether IFN-*γ* and IL-4 expression correlates closely with GD relapse remains unclear.

IL-17 is produced by Th17 cells and can enhance certain inflammatory cytokines, such as IL-1 and tumor necrosis factor alpha (TNF-*α*), and promote the migration of leukocytes to inflammatory sites [17]. Increased IL-17 expression is widely observed in various autoimmune diseases. Furthermore, the IL-17 concentration is closely associated with disease activity in patients with systemic lupus erythematosus (SLE) [18]. Recent studies have discovered higher Th17 lymphocyte levels in GD patients than healthy controls, and intractable GD shows a higher Th17 cell percentage than remittent GD does [9]. The findings in the present study did not reconcile with the reports mentioned above but are congruent with other investigations [14]. Previous data have also indicated a close correlation between GD pathogenesis and Th17 cells [19]. Another study conducted in a mouse GD model revealed that increased IL-17 expression and reduced Treg cells are possibly involved in the pathogenesis of GD [20]. Recent data have indicated the pathophysiological role of IL-17 in Graves' ophthalmopathy development due to the presence of elevated IL-17 and the correlation of IL-17 with clinical activity in GO patients [21]. Significantly higher expression levels of IL-17 protein and IL-17 mRNA were observed in GD and euthyroid GD in comparison with healthy controls by Zheng et al. [22], which suggests IL-17 to be a pathogenic factor for GD. Furthermore, many studies have proved the critical role of IL-17 in other autoimmune diseases, such as the development of experimental autoimmune encephalomyelitis [23] and collagen-induced arthritis [24]. Th17 cells are the main source of the IL-17 cytokine, and the critical role of Th17 cells in the autoimmune system might be a possible explanation for the predictive role of IL-17 in GD relapse.

We have to acknowledge some limitations in our study. First, although this study is prospective in design, no intervention strategy is applicable. Second, the duration of follow-up is relatively short. Furthermore, the mechanisms underlying how elevated IL-17 can serve as a predictor for GD relapse remains unclear. Finally, Th17 cells are not the only source of IL-17. CD8+ T cells, natural killer T cells, and lymph tissue inducer cells among others can also secrete IL-17 [25].

In conclusion, this present study indicates that elevated IL-17 expression at cessation is a predictor for GD relapse within 12 months. However, the role of IL-17 in GD remains undefined, and more relevant evidence is required to elucidate its role in GD development.

## Figures and Tables

**Table 1:** Clinical features of GD patients with or without relapse.

Parameters	Relapse		*p* value
	Yes (*n* = 45)	No (*n* = 72)	
Age at diagnosis (year)	45.3 ± 11.4	40.8 ± 10.3	0.029^∗^
Gender (*n*, %)			0.65
Male	9 (20.0%)	17 (23.6%)	
Female	36 (80.0%)	55 (76.4%)	
Smokers (*n*, %)	7(15.6%)	10(13.9%)	0.80
Graves' ophthalmopathy	20(44.4%)	18(25.0%)	0.029^∗^
Thyroglobulin (Tg, μg/L)	51.7 ± 35.9	46.8 ± 33.2	0.45
Thyroglobulin antibody positivity (%)	12(26.7%)	26(36.1%)	0.29
Anti-TPO (IU/L)	121.5 ± 98.7	94.5 ± 69.1	0.085
Duration of ATD therapy (months)	20.2 ± 5.2	19.5 ± 6.8	0.56
ATD drugs (*n*, %)			0.65
Methimazole	36 (80.0%)	60 (83.3%)	
Propylthiouracil	9 (20.0%)	12 (16.7%)	
Time to normalization of TSH (months)	5.8 ± 2.1	4.6 ± 2.4	0.007^∗^
Time to normalization of fT4 (months)	2.2 ± 1.2	1.6 ± 0.9	0.003^∗^
Thyroid function tests at diagnosis			
fT4 (pmol/L)	31.6 ± 15.1	30.8 ± 14.7	0.72
TT3 (nmol/L)	5.2 ± 1.3	4.8 ± 1.6	0.16
TSH (mIU/L)	0.06 ± 0.05	0.06 ± 0.04	1.00
TRAb (IU/L)	7.7 ± 5.8	6.7 ± 4.8	0.31
TSAb (%)	422.1 ± 172.4	482.3 ± 201.5	0.10
Thyroid function tests at cessation			
fT4 (pmol/L)	17.5 ± 2.9	16.8 ± 3.6	0.27
TT3 (nmol/L)	2.2 ± 0.5	2.3 ± 0.6	0.35
TSH (mIU/L)	2.5 ± 1.4	3.2 ± 1.5	0.013^∗^
TRAb (IU/L)	1.2 ± 0.8	0.8 ± 0.5	0.001^∗^
TSAb (%)	214.4 ± 114.8	218.5 ± 109.7	0.85

GD: Graves' disease; ATD: antithyroid drug; Anti-TPO: antithyroid peroxidase antibody; fT4: free thyroxine; TT3: total triiodothyronine; TSH: thyrotropin-stimulating hormone; TRAb: thyrotropin receptor antibody; TSAb: thyroid stimulatory antibody. *p* values were calculated by Student's *t*-test, Mann–Whitney *U* test, and chi-squared test. ^∗^*p* < 0.05.

**Table 2:** T helper cell cytokines at diagnosis/cessation in GD patients with or without relapse.

Laboratory tests	Relapse		*p* value
	Yes (*n* = 45)	No (*n* = 72)	
Cytokines at diagnosis			
IL-4 (pg/mL)	21.1 ± 5.8	19.9 ± 6.1	0.29
IL-17 (pg/mL)	10.9 ± 3.7	10.1 ± 3.2	0.22
IFN-*γ* (pg/mL)	130.4 ± 65.4	124.5 ± 55.8	0.60
Cytokines at cessation			
IL-4 (pg/mL)	15.1 ± 5.9	12.8 ± 4.7	0.022^∗^
IL-17 (pg/mL)	8.9 ± 3.1	7.1 ± 2.7	0.001^∗^
IFN-*γ* (pg/mL)	122.4 ± 54.9	118.9 ± 51.7	0.73

GD: Graves' disease; IL-4: interleukin-4; IL-17: interleukin-17; IFN-*γ*: interferon-*γ*. *p* values were calculated by Student's *t*-test. ^∗^*p* < 0.05.

**Table 3:** Risk factors for GD relapse by univariate and multiple Cox proportional hazard analysis.

Parameters	Univariate		Multivariate	
	HR (95% CI)	*p* value	HR (95% CI)	*p* value
Age at diagnosis	2.07 (1.11–3.55)	0.021^∗^	0.77 (0.34–1.55)	0.44
Graves' ophthalmopathy	1.17 (1.03–1.29)	0.014^∗^	1.01 (0.97–1.04)	0.52
Time to normalization of TSH	2.11(0.45–6.12)	0.27		
Time to normalization of fT4	1.17(0.31–4.68)	0.75		
TSH at cessation	0.98(0.94–1.04)	0.54		
TRAb at cessation	3.12 (1.06–8.78)	0.042^∗^	1.11 (0.89–1.33)	0.23
IL-4 at cessation	1.07(0.29–3.45)	0.13		
IL-17 at cessation	1.93 (1.16–3.11)	0.011^∗^	3.04 (1.14–7.67)	0.021^∗^

GD: Graves' disease; TSH: thyrotropin-stimulating hormone; fT4: free thyroxine; TRAb: thyrotropin receptor antibody; IL-4: interleukin-4; IL-17: interleukin-17; CI: confidence interval; HR: hazard ratio. ^∗^*p* < 0.05.
